# Renal expression and urinary excretion of liver‐type fatty acid‐binding protein in cats with renal disease

**DOI:** 10.1111/jvim.15721

**Published:** 2020-02-22

**Authors:** Masaaki Katayama, Keiichi Ohata, Tamako Miyazaki, Rieko Katayama, Nobuko Wakamatsu, Misa Ohno, Tetsuro Yamashita, Tsuyoshi Oikawa, Takeshi Sugaya, Masao Miyazaki

**Affiliations:** ^1^ Faculty of Agriculture, Cooperative Department of Veterinary Clinical Medicine Iwate University Morioka, Iwate Japan; ^2^ CMIC Holdings Co., Ltd Tokyo Japan; ^3^ Faculty of Agriculture, Department of Biological Chemistry and Food Sciences Iwate University Morioka, Iwate Japan; ^4^ Department of Pathobiological Sciences Louisiana State University School of Veterinary Medicine Baton Rouge Louisiana

**Keywords:** acute kidney injury, biomarker, chronic kidney disease, kidney

## Abstract

**Background:**

Liver‐type fatty acid‐binding protein (L‐FABP) is a biomarker for early detection of renal disease in humans. Liver‐type fatty acid‐binding protein is cytotoxic oxidation products secreted from proximal tubules under ischemia and oxidative stress.

**Objective:**

To examine renal expression and quantify urinary excretion of L‐FABP in catswith renal disease.

**Animals:**

One hundred and thirty‐four client‐owned cats including 34 cats with serum creatinine (sCre) values >1.6 mg/dL and 10 other cats that died in clinics.

**Methods:**

Tissue expressions of L‐FABP were examined by reverse transcription polymerase chain reaction and Western blotting. Urinary L‐FABP (uL‐FABP) and serum L‐FABP (sL‐FABP) levels were determined by enzyme‐linked immunosorbent assay. Anti‐liver‐type fatty acid‐binding protein antibody immunostained renal sections.

**Results:**

Feline kidneys express L‐FABP. Strong L‐FABP signals were observed in the lumens of proximal tubular cells in 5 cats with high uL‐FABP excretion, but not in 5 cats with low uL‐FABP excretion. In 9 normal cats, uL‐FABP index was <1.2 μg/g urinary creatinine (uCre). High uL‐FABP indexes (>10.0 μg/g uCre) were detected in 7 of 100 cats with low sCre (<1.6 mg/dL) and 18 of 44 cats with high sCre (>1.6 mg/dL). There was a weak correlation between L‐FABP index and sCre, serum symmetric dimethylarginine (SDMA), or blood urea nitrogen (BUN), and these correlation coefficients were increased by analyzing only data of cats with sCre >1.6 mg/dL. There was a weak correlation between u L‐FABP index and sL‐FABP in all tested cats, but not in cats with high sCre.

**Conclusions and Clinical Importance:**

This study demonstrates correlations between L‐FABP and current renal biomarkers for chronic kidney disease in cats, such as sCre and SDMA. Liver‐type fatty acid‐binding protein may be a potential biomarker to predict early pathophysiological events in feline kidneys.

**Abbreviations**
AKI: acute kidney injury
CKD: chronic kidney disease
Cre: creatinine
ELISA: enzyme‐linked immunosorbent assay
GFR: glomerular filtration rate
HRP: horseradish peroxidase
IRIS: International Renal Interest Society
L‐FABP: liver‐type fatty acid binding protein
NAG: *N*‐acetyl‐β‐d‐glucosaminidase
SDMA: symmetric dimethylarginine
sL‐FABP: serum L‐FABP
RT‐PCR: reverse transcription‐polymerase chain reaction
uL‐FABP: urinary L‐FABP
USG: urine specific gravity

## 1 INTRODUCTION

Renal diseases are common in domestic cats, especially elderly animals.^1^, ^2^, ^3^ Chronic kidney disease (CKD), which results in uremia at the end stages, is a leading cause of illness and death in elderly cats. The development of CKD was reported in 31% of clinically healthy cats >9 years of age.^4^ The incidence of CKD in cats is 15% at >15 years of age,^2^ and more recently as 27% at >10 years of age.^5^ In addition to CKD, acute kidney injury (AKI) is also an important renal disease in cats, with a case fatality rate exceeding 50% despite treatment.^6^, ^7^ Therefore, it is crucial to diagnose CKD and AKI in the early stages to prevent their progression and for clinical management.

Proteinuria is an early indicator used to identify renal dysfunction in animals.^8^ Some specific proteins are present in the urine in cases of renal disease. For example, retinol binding protein^9^ and cystatin C^10^ levels are increased by dysfunction of proximal tubular reabsorption in cats. In contrast, Tamm‐Horsfall protein excretion decreases in cats associated with decreases in its production by damage to distal tubular cells.^11^ Carboxylesterase 5A (also known as cauxin), which produces a sex pheromone,^12^ is a major urinary protein in normal healthy cats^13^ and its excretion is decreased in cats with CKD due to decrease of number of proximal tubular cells expressing it.^14^, ^15^, ^16^, ^17^ The increase of urinary excretion of these proteins usually results from functional, structural, or both damages in the glomerulus and renal tubules.

Urinary excretion of liver‐type fatty acid binding protein (L‐FABP) has been clinically recognized as a useful biomarker for monitoring early detection of AKI and CKD in humans.^18^, ^19^ In humans, L‐FABP is expressed in renal proximal tubular cells in addition to the liver, pancreas, and small intestine.^20^ The functions of L‐FABP include binding to and transporting fatty acids to the mitochondria or peroxisomes, where they are β‐oxidized, and participation in intracellular fatty acid homeostasis.^21^ Liver‐type fatty acid binding protein is excreted in urine due to ischemia (or low capillary blood flow) and oxidative stress on the renal tubules before the progression of renal damage.^22^, ^23^ In the absence of renal diseases, L‐FABP secreted from the liver into the blood crosses the glomerular barrier and then is reabsorbed by the proximal tubular cells, resulting that such L‐FABP hardly appears in urine, in contrast, in the case of hepatitis fulminant or hepatorenal syndrome, approximately 15% of urinary L‐FABPs (uL‐FABPs) originate in the liver (CMIC Holdings, unpublished data).

Renal expression of L‐FABP is species specific, and the expression of L‐FABP is genetically suppressed in the kidneys of rodents, such as mice.^24^ Therefore, it is necessary to determine whether L‐FABP is expressed in the proximal tubular epithelial cells of the cat kidney as well as humans. The aim of this study was to investigate L‐FABP expression in the cat kidney and the relationship between levels of L‐FABP excretion and its renal distribution. We also examined the correlations between uL‐FABP and current renal biomarkers in cats with renal diseases. These results will improve our understanding of the pathophysiological significance of L‐FABP in cats.

## 2 MATERIALS AND METHODS

### 2.1 Animals and sample collection

Urine and blood samples were collected from 134 cats (24 intact males, 28 castrated males, 36 intact females, and 46 spayed females, between 5 months and 21 years old), which had been treated at veterinary clinics between 2005 and 2018. Samples of 9 of the 134 cats were collected in our previous study.^15^ Urine and kidney tissue samples were collected from 10 cats that died in veterinary clinics in the United States and then used for postmortem histopathological examination. We used normal kidney and liver tissues of a male cat, which were sampled in our previous study and stored at −80°C.^25^ Urine samples were collected by cystocentesis using 23‐gauge needles. Blood (0.5 mL) was collected from the cephalic vein using a 24‐gauge needle. All urine and blood samples were centrifuged at ×500 for 5 minutes, and the supernatants were stored at −30°C until biochemical analyses, which had been done within 8 months after sampling. This study was performed in accordance with local animal ethics guidelines and was approved by the Animal Research Committee of our university. All feline owners approved of providing the urine, blood, and kidney samples of their cats for this study.

### 2.2 Reverse transcription‐polymerase chain reaction

Total RNA was extracted from the kidneys and liver of an intact male cat using TRIzol reagent (Gibco BRL, Grand Island, NY). cDNAs were generated from 1 μg of total RNA from the kidney and liver using ReverTra Ace (Toyobo, Osaka, Japan) with oligo(dT)_15_ primer. Polymerase chain reaction (PCR) was performed with KOD‐Plus (Toyobo) using the cDNAs and the following primers: L‐FABP (GenBank accession no. M3WR74): forward, 5′‐GAACTTCTCCGGCAAGTACCA‐3′; reverse, 5′‐ATGTCGCCATTGAGTTCGGT‐3′; and glyceraldehyde‐3‐phosphate dehydrogenase (GAPDH, GenBank accession no. AB038241): forward, 5′‐ACCACAGTCCATGCCATCAC‐3′; reverse, 5′‐TCCACCACCCTGTTGCTGTA‐3′.

### 2.3 Western blotting

Approximately, 0.5 g of frozen kidney and liver of a normal cat were homogenized in 5 mL of 0.1 M Tris buffer (pH 7.4) containing protease inhibitors (Roche Diagnostics, Penzberg, Germany) on ice. The supernatant (30 μg), after centrifugation at 15000 rpm at 4°C for 20 minutes, and cat urine (aliquot 10 μL) were resolved by Sodium dodecyl sulfate‐polyacrylamide gel electrophoresis (SDS‐PAGE, 15% gel) under reducing conditions, and then proteins were transferred onto polyvinylidene difluoride membranes by semi‐dry blotting (2 mA/cm^2^). The membranes were blocked with 2% skim milk in tris buffered saline with tween 20 (TBS‐T, 10mM Tris‐HCl (pH 7.4), 150 mM NaCl, 0.05% Tween 20), and incubated with anti‐L‐FABP monoclonal antibody (clone 2, CMIC Holdings, diluted 1:2000) for 1 hour as primary antibodies. The membranes were then incubated with horseradish peroxidase (HRP)‐conjugated anti‐mouse IgG antibody (Abcam) for 1 hour, and the peroxidase activity was detected using an enhanced chemiluminescence kit (ECL Plus; GE Healthcare, Waukesha, WI). Signals were detected using a LAS 2000 image analyzer (Fuji film, Tokyo, Japan).

### 2.4 Enzyme‐linked immunosorbent assay

Urinary L‐FABP and serum L‐FABP (sL‐FABP) concentrations were measured using a 2‐step sandwich enzyme‐linked immunosorbent assay (ELISA) to the manufacturer's instructions (Feline L‐FABP ELISA kit; CMIC Holdings). The detectable range of L‐FABP was ≥0.49 ng/mL in the ELISA kit. Urinary L‐FABP levels were normalized relative to urinary creatinine (uCre) concentration to avoid variations caused by urinary volume and expressed as uL‐FABP index. uCre levels were measured by Folin's method.^26^

### 2.5 Immunohistochemistry

Paraffin sections of feline kidneys were prepared as described previously (Miyazaki et al., 2003). Endogenous peroxidase was blocked with 0.3% H_2_O_2_ in methanol for the detection of HRP activity. Deparaffinized sections were incubated with 10% goat serum in PBS at room temperature for 1 hour and then with anti‐human L‐FABP monoclonal antibody (clone 2, CMIC Holdings, diluted 1:200) at room temperature for 1 hour. After treatment with HRP‐labeled polymer‐conjugated anti‐mouse IgG antibody (EnVision+, peroxidase, rabbit; Dako, Glostrup, Denmark) for 30 minutes at room temperature. The sections were treated with diaminobenzidine to visualize the antigen‐antibody reaction. Finally, the sections were counterstained with hematoxylin and observed under a light microscope.

### 2.6 Others

Serum symmetric dimethylarginine (SDMA) was measured by IDEXX Laboratories (Tokyo, Japan). The correlation between density of the L‐FABP band detected by Western blotting and uL‐FABP concentration measured by ELISA were determined by Spearman's rank correlation analysis. Serum L‐FABP, uL‐FABP/uCre, sCre, BUN, and SDMA values were logarithmically transformed for statistical analyses of Spearman's rank correlation analysis. Statistical significances of uL‐FABP/uCre between international renal interest society (IRIS) stages II, III, and IV were tested using nonparametric Wilcoxon matched‐pair signed ranks tests. All statistical analyses were performed using JMP12.2.0 statistical software (SAS Institute, Cary, North Carolina). In all analyses, results were considered statistically significant at *P* < .05.

## 3 RESULTS

### 3.1 Liver‐type fatty acid‐binding protein expression in feline kidneys

**Figure 1:**
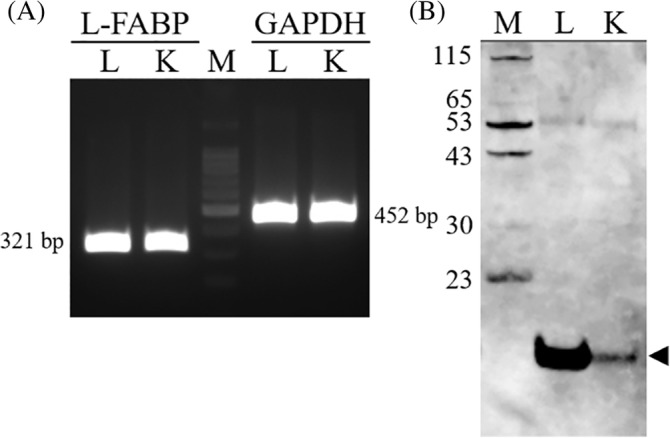
Expression of L‐FABP in feline kidneys. A, RT‐PCR for amplification of feline L‐FABP and GAPDH was performed with kidney and liver mRNAs from a normal male cat. The feline L‐FABP gene (amplicon size 321 bp) and GAPDH gene (amplicon size 452 bp) were amplified from renal and liver cDNAs using specific primers. B, Western blotting of proteins (aliquots of 30 μg) extracted from cat kidneys and livers was performed using anti‐L‐FABP antibodies. An arrow indicates an signal of 14‐kDa L‐FABP. L, liver, K, kidney; M, marker (Figure 1A, 100‐bp ladder marker). L‐FABP, liver‐type fatty acid‐binding protein; RT‐PCR, reverse transcription polymerase chain reaction

We first analyzed the expression of L‐FABP mRNA in cat kidneys by RT‐PCR with the GAPDH amplicon (452 bp) as a positive control. Liver‐type fatty acid‐binding protein amplicons (321 bp) were detected in the cat kidneys as well as cat livers (Figure 1A). To analyze L‐FABP expression at the protein level, Western blotting analysis was carried out using anti‐human L‐FABP monoclonal antibodies. As the whole amino acid sequence of L‐FABP is conserved between human and cat at high similarity (91%), it was expected that monoclonal antibodies would cross‐react with feline L‐FABP. Western blotting detected L‐FABP proteins in both the feline kidney and liver (Figure 1B). Signals of L‐FABP were stronger in the liver than the kidney, indicating that the expression level of L‐FABP was higher in the liver than the kidney in cats. These results indicated that cat and human kidneys express L‐FABP similarly.

### 3.2 Urinary excretion of L‐FABP in cats

**Figure 2:**
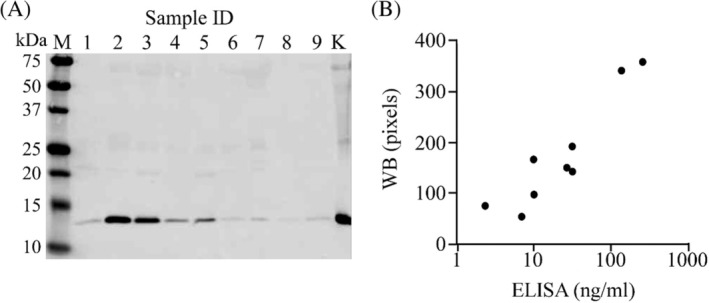
Urinary excretion of L‐FABP in cats. A, Western blotting of urine (aliquots of 10 μL) collected from 9 cats with CKD was performed using anti‐L‐FABP antibody. Other detail information of the 9 cats is described in Table S1. B, Correlation between urinary L‐FABP contents quantified by L‐FABP ELISA and density of the L‐FABP band detected in Figure 2A. CKD, chronic kidney disease; ELISA, enzyme‐linked immunosorbent assay; L‐FABP, liver‐type fatty acid‐binding protein

Preliminary studies were carried out to examine L‐FABP excretions in cats urine using samples collected from 9 cats with CKD. They had high sCre concentrations (2.5‐11.3 mg/dL, median 6.1 mg/dL). Western blotting using anti‐L‐FABP antibody detected a single band corresponding to 14‐kDa L‐FABP in all urine samples as well as protein extracts of the normal feline kidney (Figure 2A). Especially, high levels of L‐FABP excretion were observed in 4 of the 9 cats with CKD (lanes 2‐5). Because most of their urine specific gravity (USG) was under 1.010 (Table S1), uL‐FABP values were normalized to the uCre concentrations. In the 9 cats with CKD, the uL‐FABP/uCre ranged from 97.60 to 6308.25 μg/g uCre (median 225.91 μg/g uCre). The results indicated that L‐FABP is excreted in the urine of cats with CKD.

Next, the correlation was examined between density of the L‐FABP band detected by Western blotting and uL‐FABP contents quantified by the L‐FABP ELISA kit. As we confirmed a significant correlation between these values (Spearman's rho: 0.8167; *P* = .007, Figure 2B), ELISA was used for measurement of L‐FABP in the following experiments.

### 3.3 Urinary excretion and renal distributions of L‐FABP in 10 cats

**Figure 3:**
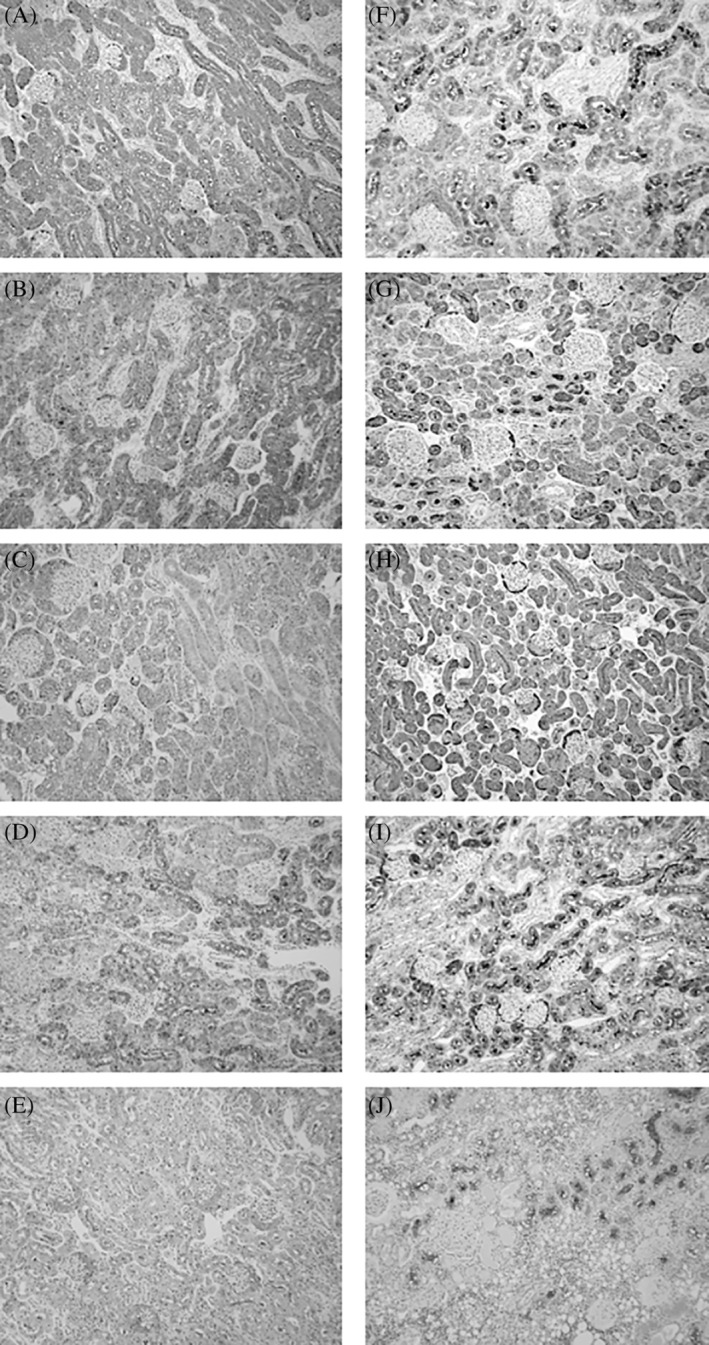
Representative immunohistochemical images of L‐FABP in paraffin‐embedded renal sections of 10 cats. A‐E and F‐J are renal sections of 5 cats with low urinary L‐FABP index and 5 cats with high uL‐FABP index, respectively. Their individual information, urinary L‐FABP indexes, pathological diagnosis, and clinical history are described in Table S2. L‐FABP, liver‐type fatty acid‐binding protein; uL‐FABP, urinary L‐FABP

We collected both renal tissue and urine samples from 10 cats that died in veterinary clinics by necropsy. Urinary L‐FABP index, pathological diagnosis, and clinical history were shown in Table S2. They did not have hepatopathy, enteropathy, or pancreatitis, except for a cat who had hepatocellular vacuolar degeneration in addition to hypertrophic cardiomyopathy. The uL‐FABP indexes of 5 cats (cases 1‐5) were under 10 μg/g uCre and those of the other 5 cats (cases 6‐10) were >100 μg/g uCre. We compared tissue localization of L‐FABP between the low uL‐FABP and high uL‐FABP groups. There were no lesions detected in the kidneys of cats with low uL‐FABP. In these cases, L‐FABP signals were mainly localized in the cytoplasm of proximal tubular epithelial cells (Figure 3A‐E). In contrast, strong L‐FABP signals were observed not only in the cytoplasm but also in the lumens of the proximal tubular cells of the 5 cats with high uL‐FABP (Figure 3F–J). In a cat (case no. 10), tubular cells had disappeared in the damaged area where glomerular and tubular epithelial cells showed multifocal severe fibrotic changes (Figure 3J). Although no L‐FABP signals were observed in these damaged areas, strong L‐FABP signals were observed in the lumens of the proximal tubules in the normal area.

### 3.4 Correlation analyses between uL‐FABP and sL‐FABP

**Figure 4:**
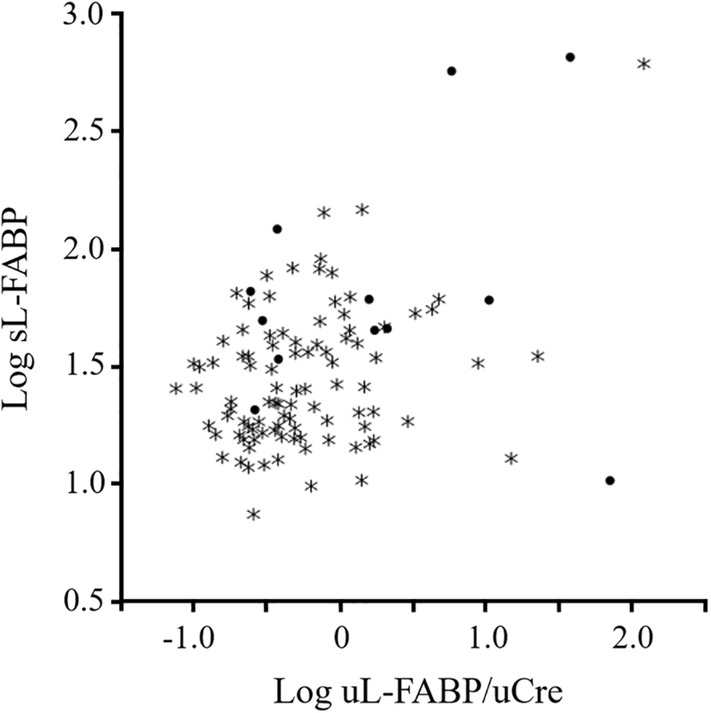
Correlations between serum and urinary L‐FABP in cats. The correlation between Log serum L‐FABP and Log urinary L‐FABP index was examined in 108 cats including 12 cats with high sCre (>1.6 mg/dL). Asterisks indicate cats with low sCre (<1.6 mg/dL). L‐FABP, liver‐type fatty acid‐binding protein; sCre, serum creatinine

The serum concentrations of L‐FABP were measured using the L‐FABP ELISA kit in 108 of 134 cats, which contained 12 cats whose sCre was >1.6 mg/dL (IRIS ≥ II). We could not measure sL‐FABP contents in other 26 cats because of the lack of enough volume of serum for analysis. The concentration of sL‐FABP ranged from 7.33 to 655.94 ng/mL (median 25.945 ng/mL). Spearman's rank correlation coefficient analysis showed that there was a significant weak correlation between Log uL‐FABP index and Log sL‐FABP in all 108 cats (Spearman's rho: 0.2510; *P* = .0088, Figure 4), but not in the 12 cats with high sCre (Spearman's rho: 0.0559; *P* = 0.8629). In case no. 124 and 62 (Table S1), sL‐FABPs were same values (10.2 ng/mL), but uL‐FABP indexes were markedly different values (71.97 μg/g uCre) and (1.44 μg/g uCre), respectively. In other 2 cases with high sL‐FABP values (>600 ng/mL), uL‐FABP index was higher in case no. 11 (122.79 μg/g uCre) than case no. 111 (38.47 μg/g uCre).

### 3.5 Correlation analyses between uL‐FABP and sCre or SDMA

We examined the correlations between uL‐FABP index and sCre, BUN, or SDMA in 134 cats of which detail information is shown in Table S1. Serum SDMA is derived from intranuclear methylation of l‐arginine by protein arginine methyltransferase and primarily eliminated by renal excretion, suggesting that SDMA is a potential endogenous marker of glomerular filtration rate (GFR).^27^ Nine of the 134 cats were allocated into the normal group because they were young (under 6 years old) and healthy with low sCre (under 1.2 mg/dL), SDMA (<14 μg/dL), and alanine transaminase (ALT, under 53 U/L). In the normal group, the uL‐FABP/uCre ranged from 0.16 to 1.19 μg/g uCre (median 0.41 μg/g uCre). Eighteen of 34 cats with sCre values >1.6 mg/dL had high uL‐FABP/uCre (>10.0 μg/g uCre), but the other 16 cats did not (uL‐FABP/uCre: 0.25‐6.44 μg/g, median 1.435 μg/g uCre). On the other hand, 7 of 100 cats with sCre values under 1.6 mg/dL had high L‐FABP/uCre (>10.0 μg/g uCre).

**Figure 5:**
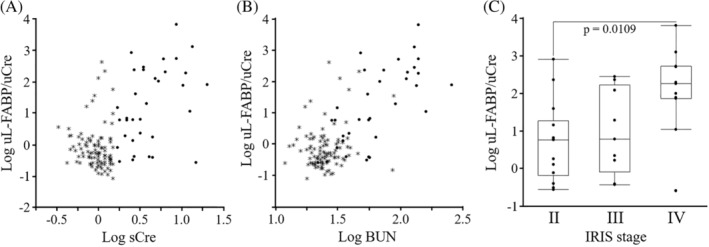
Correlations between urinary L‐FABP, sCre, or BUN in cats. The correlation between Log uL‐FABP index and Log sCre (A) or Log BUN (B) was examined in 134 cats including 24 cats with high sCre (>1.6 mg/dL). Asterisks indicate cats with low sCre (<1.6 mg/dL). C. Log uL‐FABP indexes are shown inbox and whisker plot, and their significant differences between IRIS stages were evaluated by nonparametric Wilcoxon matched‐pair signed ranks tests. BUN, blood urea nitrogen, L‐FABP, liver‐type fatty acid‐binding protein; sCre, serum creatinine; uL‐FABP, urinary L‐FABP

Spearman's rank correlation coefficient analysis showed that there are significant correlation between Log uL‐FABP/uCre and Log sCre (Spearman's rho: 0.2880; *P* = .0007, Figure 5A) and between Log uL‐FABP/uCre and Log BUN (Spearman's rho: 0.4420; *P* = .0001, Figure 5B). These correlation coefficients were increased between Log uL‐FABP/uCre and Log sCre (Spearman's rho: 0.3944; *P* = .0210) and between Log uL‐FABP/uCre and Log BUN (Spearman's rho: 0.6637; *P* < .0001) by analyzing data only of 34 cats whose sCre was >1.6 mg/dL.

Thirty‐four of the 134 cats was diagnosed as azotemic CKD based on sCre concentration >1.6 mg/dL in conjunction with USG < 1.035.^28^ According to International Renal Interest Society (IRIS) guidelines,^29^ 14, 9, and 11 cats were classified into IRIS stages II, III, and IV, respectively. Urinary L‐FABP /uCre was significantly higher in the 11 cats with IRIS stage IV than the 14 cats with IRIS stage II (Figure 5C). Urinary L‐FABP/uCre >10.0 μg/g uCre was observed in 4 of 14 (29%) cats with IRIS stage II, 4 of 9 (44%) cats with IRIS stage III, and 10 of 11 (91%) cats with IRIS stage IV, respectively.

**Figure 6:**
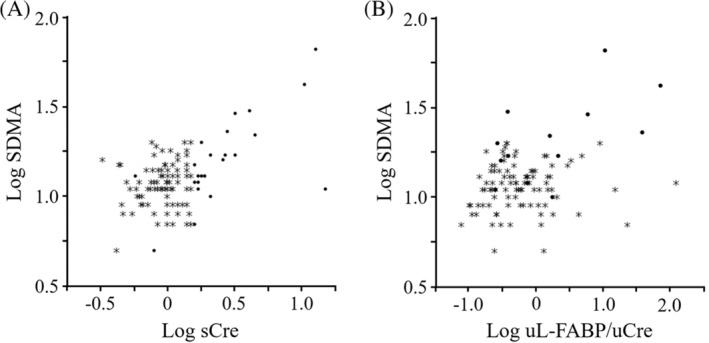
Correlations between serum SDMA and urinary L‐FABP or sCre in cats. The correlation between Log serum SDMA and Log sCre (A) or urinary L‐FABP index (B) was examined in 106 cats including 13 cats with high sCre (>1.6 mg/dL). Asterisks indicate cats with low sCre (<1.6 mg/dL). L‐FABP, liver‐type fatty acid‐binding protein; sCre, serum creatinine; SDMA, serum symmetric dimethylarginine

Serum SDMA concentrations were measured in 106 of 134 cats, which contained 13 cats of which sCre was >1.6 mg/dL. We could not measure SDMA in other 28 cats because of the lack of enough serum volume. There is a significant positive correlation between Log sCre and Log SDMA (Spearman's rho: 0.2924; *P* = .0024, Figure 6A) and between Log uL‐FABP/uCre and Log SDMA (Spearman's rho: 0.2018; *P* = .0308, Figure 6B) in 106 cats. These correlation coefficients were not significant between Log sCre and Log SDMA (Spearman's rho: 0.4945; *P* = 0.0858) and increased between Log uL‐FABP/uCre and Log SDMA (Spearman's rho: 0.5557; *P* = 0.0486) by analyzing data only of 13 cats whose sCre was >1.6 mg/dL. Three of 69 cats with serum SDMA values <14 μg/dL had high L‐FABP/uCre (15.2, 23.1, and 122.8 μg/g uCre), whereas the other 66 cats did not (L‐FABP/uCre: 0.1‐4.4 μg/g uCre, median 0.5 μg/g uCre). On the other hand, only 3 of 37 cats with serum SDMA values >14 μg/dL had high L‐FABP/uCre (10.72 and 38.47, 71.97 μg/g uCre).

## 4 DISCUSSION

In the present study, we examined renal expression and urinary excretion of L‐FABP in cats. The major findings were (1) L‐FABP expression in cat kidneys, (2) the distribution of L‐FABP in the renal tubular lumen in cats with high uL‐FABP excretion but not in cats with low uL‐FABP excretion, and (3) the presence of cats with high uL‐FABP excretion without abnormal values in other renal biomarkers, such as sCre and SDMA. In addition to human, L‐FABP expression in the kidneys was reported only in rats.^30^ Previous studies examined urinary excretion of L‐FABP in dogs^31^ and pigs,^32^ but they did not show the renal expression of L‐FABP. Because L‐FABP expresses not only kidneys but also other tissues such as the liver, pancreas, and small intestine,^20^ it is necessary to examine the renal expression of L‐FABP in each species. This report demonstrates that L‐FABP is physiologically expressed in the kidneys of animals, as seen in humans.

Liver‐type fatty acid‐binding protein signals were detected in the renal tubular lumen in 5 cats with high uL‐FABP/uCre. Such renal distributions of uL‐FABP were not observed in the other 5 cats with low uL‐FABP/uCre. These results show the relevance between levels of uL‐FABP excretion and its renal localization in cats. In humans, uL‐FABP level is mostly determined by proximal tubular injury rather than liver damage,^33^, ^34^ and sL‐FABP is not correlated with uL‐FABP in patients with CKD.^35^ The increase in L‐FABP expression in the proximal tubules and subsequent secretion of L‐FABP into the tubular lumen occurs in transgenic mice expressing human L‐FABP in the kidneys when they exhibit renal failure.^19^, ^36^ Therefore, we suggest that uL‐FABP is mainly derived from proximal tubular cells in cats.

This study showed no significant correlation between sL‐FABP and uL‐FABP index in cats with high sCre; some cats with high sL‐FABP exhibited low uL‐FABP/uCre, in contrast the other cats with low sL‐FABP did high uL‐FABP/uCre. On the other hand, there was a cat (Table S1, case no. 11) that had both high sL‐FABP and uL‐FABP/uCre values under normal values of sCre (1.0 mg/dL) but high values of ALT (238.0 U/L). Based on a USG value (1.022), it is suggested that the renal function for concentrating urine decline in the cat, resulting that not only leak of sL‐FABP but also the renal secretion of L‐FABP might be because of high uL‐FABP/uCre in such case. It can be distinguished through joint use with other biomarkers, such as sCre, SDMA, ALT, and USG, whether the increase of uL‐FABP is caused for renal stresses.

In both analyses of urinary excretion and renal distributions of L‐FABP in 10 cats, 2 clinical cases showed high uL‐FABP index without pathohistological damage to the kidneys. Previous studies indicated that urinary excretion of L‐FABP increases even before the occurrence of structural damage of proximal tubules with ischemia and oxidative stress.^33^, ^37^ Their high uL‐FABP index could represent evidence of proximal tubular cells with ischemia or oxidative stress even if they did not have structural renal damage. On the other hand, this study found 18 cats with high sCre values (>1.6 mg/dL) but low uL‐FABP index (uL‐FABP/uCre <10). We suspected that the 18 cats lost normal renal function because of renal structural damages but had few proximal tubules under conditions of ischemia or oxidative stress in nondamaged areas. We strongly suggest that uL‐FABP excretion is increased before renal structural and functional damages in cats.

We measured uL‐FABP index in 134 cats including 9 normal cats and 34 cats with high sCre concentrations (>1.6 mg/dL). One limitation of the present study was that only a single measurement of uL‐FABP was taken at 1 time point. The 9 cats comprising the normal group showed lower uL‐FABP index (0.16‐1.19 μg/g uCre, median 0.41 μg/g uCre). Although uL‐FABP should be measured in larger numbers of cats, the cutoff values of uL‐FABP for monitoring of AKI and CKD in cats would be 4 μg/g uCre, which is lower than in humans whose values are <8.4 μg/g uCre.^38^

The present study suggests that it will be valuable to find cats with high values of uL‐FABP index without increases in other renal biomarkers for diagnosis of early AKI in cats. There was a weak correlation, but significantly between uL‐FABP and SDMA in 106 cats, and between uL‐FABP and sCre in 134 cats, in addition to the positive correlation between SDMA and sCre as described in previous reports.^39^, ^40^ To predict early pathophysiological events in feline kidneys, we are now focusing on cats that exhibit high L‐FABP/uCre (>10 μg/g uCre) but low sCre (<1.6 mg/dL) and low serum SDMA (<14 μg/dL). This might be because the mechanisms underlying increases in uL‐FABP excretion are different with sCre or SDMA. sCre and SDMA are biomarkers to assess renal function and they increase when GFR has declined. In contrast, L‐FABP excretion reflects the tubular response due to ischemia and oxidative stress, and increases from the early stages of pathological events before structural damage of the glomerular, tubular, and both cells.^33^, ^37^ Other biomarkers, such as urinary albumin^41^ and *N*‐acetyl‐β‐d‐glucosaminidase (NAG),^16^ are used to diagnose early stages of AKI and CKD in cats. However, urinary albumin and NAG increase with progression of glomerular and tubular injuries, respectively. On the other hand, a recent study indicated that urinary heat‐shock protein‐72 level increases under conditions of cellular stress in the feline kidney, suggesting that it could be useful as a biomarker for early diagnosis of AKI and CKD in cats.^42^ This suggests that proteins that increase pathophysiological events but not structural and functional damage to the kidney might be suitable as biomarkers for early prediction of renal diseases in cats. Therefore, we expect to find early stages of AKI and CKD from cats with high uL‐FABP index.

It is crucial to diagnose the early stages of CKD and AKI in cats for their clinical managements. Ischemic damage is a common cause of AKI in human patients.^43^ AKI and CKD are bidirectionally connected, as the presence of CKD represents a predisposing factor for AKI and the occurrence of AKI is a risk factor for subsequent development of CKD.^44^ In cats, AKI can be caused by toxic, ischemic, inflammatory, obstructive, or infectious insults.^6^, ^7^, ^45^ AKI initiates a cascade that results in chronic and progressive tubulointerstitial inflammation and fibrosis closely mimicking those observed in naturally occurring CKD.^46^ Although the bidirectional nature of this relationship was not investigated, AKI could be a factor in the development or progression of CKD in cats as well as in humans. Therefore, we propose that L‐FABP could predict ischemia‐reperfusion injury‐induced AKI and CKD in cats. Further studies involving measurement of uL‐FABP at several time points before diagnosis of renal diseases and long‐term monitoring of uL‐FABP with survival time in large populations are necessary to evaluate the utility of uL‐FABP as a biomarker in cats.

## 5 CONCLUSIONS

The present study indicated that L‐FABP is expressed in the kidneys of cats as in humans. We propose that L‐FABP is a potential biomarker to detect the presence of pathophysiological events in cat kidneys. Cats showing excretion of large amounts of L‐FABP into the urine even in the absence of abnormal values in other biomarkers, such as sCre and SDMA, might be at risk of developing AKI or CKD. On the other hand, considering the high prevalence rates of naturally occurring AKI and CKD in cats, they are excellent models to investigate the pathophysiological roles of L‐FABP in the kidneys.

## CONFLICT OF INTEREST

Tsuyoshi Oikawa, Keiichi Ohata, and Takeshi Sugaya are senior scientists of CMIC Holdings Co. Ltd (Tokyo, Japan), a company that produced the high sensitivity L‐FABP ELISA kits for L‐FABP analysis. No other potential conflicts of interest relevant to this article are reported.

## OFF‐LABEL ANTIMICROBIAL DECLARATION

Authors declare no off‐label use of antimicrobials.

## INSTITUTIONAL ANIMAL CARE AND USE COMMITTEE (IACUC) OR OTHER APPROVAL DECLARATION

This study was performed in accordance with local animal ethics guidelines and was approved by the Animal Research Committee of the Faculty of Agriculture of Iwate University (#A201432).

## HUMAN ETHICS APPROVAL DECLARATION

Authors declare human ethics approval was not needed for this study.

## Supporting information


**Table S1** Individual information and urinary and serum biochemical data in 134 cats, related to Figures 2, 4, 5, and 6.

**Table S2**Clinical histories and uL‐FABP index of 10 cats, related to Figure 3.
