# Supplementary material for: Renal expression and urinary excretion of liver‐type fatty acid‐binding protein in cats with renal disease
Source: J Vet Intern Med. 2020 Feb 22;34(2):761–9. doi: 10.1111/jvim.15721 (PMC7096645; doi:10.1111/jvim.15721)
Supplement: Supplementary file 2 — Table S2Clinical histories and uL‐FABP index of 10 cats, related to Figure 3. [file JVIM-34-761-s002.pdf]

Supplemental Table 2. Clinical histories and uL-FABP index of 10 cats, related to Figure 3.

Case no.	Breed	Sex	Age	B.W. (kg)	uL-FABP (ng/L)	uCre (g/L)	uL-FABP/uCre	Pathological diagnosis	Pathohistology of renal sections	Clinical data	Figure 3
1	DSH	F	1	3.8	0.14	1.43	0.1	Cardiac myofiber disarray	Normal	Heart failure for the heartworm disease	A
2	DSH	M	1	5.5	9.14	1.29	7.1	Acute thymic hemorrhage	Normal	Trauma	B
3	DSH	C	7	8	0.26	2.59	0.1	Hypertrophic cardiomyopathy and hepatocellular vacuolar degeneration	Normal	Trauma. The cat had history of leg pain and weakness. Spastic episode for a few min before death.	C
4	DSH	F	1	2.7	10.99	1.83	6.0	Pulmonary edema and mild lymphocytic myocarditis	Normal	Cardiac arrest after anesthetization for surgery	D
5	DSH	F	8M	2	0.24	0.20	1.2	Hydrocephalus brain by feline infectious peritonitis and multifocal severe lymphocytic and granulomatous encephalitis	Normal	Feline infectious peritonitis	E
6	DSH	C	2	4.5	4888.48	0.09	54926.7	Lymphoplasmacytic interstitial nephritis	Multifocal mild lymphoplasmacytic interstitial nephritis	Trauma by bites of a dog	F
7	DSH	S	5	4	55.46	0.46	121.1	Disseminated neuroendocrine tumor	Perivascular aggregation of neoplastic cells in focal adipose tissues around the renal pelvis	Neuroendocrine tumor	G
8	DSH	M	N.D	2.2	585.96	0.34	1728.5	None	Normal	The cat with no papillary light reflex and proprioceptive deficits of all four legs was found on side of road.	H
9	DSH	C	1	1	1608.32	0.04	43468.2	Bacterial pneumonia lung	Normal	Hypoglycemia, Non-regenerative anemia, Hypothermia, Sepsis, and Hypoalbuminemia.	I
10	DSH	C	14	3.5	27.86	0.12	232.2	Chronic active cholangiohepatitis and heartworm disease	Multifocal severe renal fibrosis with multifocal renal cysts	The cat was shaking and very weak when he returned from outside.	J

DSH: domestic short-haired cat, M: male, C: castrated male, F: female, S: spayed female, N.D. : no data
